# Imaging hallmarks of idiopathic intracranial hypertension and insights into pathogenesis

**DOI:** 10.3389/fradi.2025.1605777

**Published:** 2025-05-21

**Authors:** Albalooshy Basimah, Scott Scott Faro, Hsiangkuo Yuan, Kiran Talekar, Prabath Mondel, Enchao Qiu, Joga Chaganti

**Affiliations:** ^1^ Department of Radiology, Thomas Jefferson University, Philadelphia, PA, United States; ^2^ Jefferson Headache Center, Department of Neurology, Thomas Jefferson University, Philadelphia, PA, United States

**Keywords:** idiopathic intracranial hypertension, glymphatic system, magnetic resonance, diffusion tensor image analysis along the perivascular space, intracranial pressure

## Abstract

Idiopathic Intracranial hypertension (IIH), also referred to as pseudotumor cerebri, is a term used to describe increased intracranial pressure in the absence of a known identifiable secondary cause. Despite advancements of neuroimaging techniques, imaging of the pathological underpinnings in the diagnosis of IIH has been limited. Although the causation of IIH has been ascribed to increased Cerebrospinal Fluid production and disordered drainage through the dural sinuses, new evidence shows that the glymphatic system which is an alternate pathway of drainage is likely to play a pivotal role. In this review, we address the pathophysiological underpinnings in the causation of IIH and discusses characteristic anatomical imaging findings on conventional MRI and explore the role of advanced imaging techniques.

## Introduction

Idiopathic Intracranial hypertension (IIH), also referred to as pseudotumor cerebri, is a term used to describe increased intracranial pressure in the absence of an identifiable secondary cause. IIH is a rare clinical syndrome with an incidence of 0.28–3.2/100,000 people per year (1). Amongst those at highest risk, namely women of child-bearing age with a high body mass index, the incidence is 7.9–22/100,000 people per year (1). Multiple studies have shown a strong association between elevated body mass index, obstructive sleep apnoea, several endocrine disorders, and Vitamin A and tetracycline usage. Recent evidence also indicates androgen excess in IIH and, hence, a neurometabolic angle to the disease causation. In a retrospective case review, the incidence of IIH was reported as 1.56 per 100,000 in the general population, increasing to 11.90 per 100,000 in obese young women (2). The most common clinical symptom is headache, followed by visual deficits due to papilledema. Often, the diagnosis is established by the Modified Dandy Criteria and Freidman modifications consisting of an opening pressure at lumbar puncture (performed in lateral decubitus position) of over 25 cm of water in adults and >28 cm in children, with no definable etiology and normal cerebrospinal fluid (CSF) constituents (3–5).

In this review, we describe the characteristic structural findings of IIH on MRI and discuss the advanced imaging techniques to facilitate the understanding of the pathophysiological basis of IIH.

## Pathogenesis

Although the pathophysiology is largely unknown, there are indications that the pathological underpinnings for IIH appear to be at the level of dysregulated CSF production, incomplete drainage of the solutes, and neurovascular glial unit dysfunction (1).

## CSF FLOW production and drainage

CSF is produced from the blood ultrafiltrate, generated by Aquaporin-1 (AQP-1) channels in the choroid plexus and circulates through the ventricular system and cisternal spaces and enters either spinal subarachnoid space or the interstitial space of the brain and gets reabsorbed through lymphatic and venous channels. CSF serves several essential functions, such as homeostasis in the blood-CSF barrier, temperature maintenance, provision of nutrients, and regulation of the osmotic pressure (1, 6).

The movement of CSF is likely due to hydrostatic gradients initially from the production in the ventricles to the interstitial space. However, the subsequent egress of the CSF into the venous sinuses is a dynamic process facilitated by aquaporin-4 (AQP4) protein channels. This a brain-wide pathway for fluid transport, which includes the para-arterial influx of subarachnoid CSF into the brain interstitium, followed by the clearance of interstitial fluid (ISF) along large-calibre draining veins. This model assumes three main components: A: periarterial inflow of fluid from the subarachnoid spaces at the brain surface to the parenchymal interstitium, in part via the water-selective membrane channel AQP4; B: intraparenchymal flow of fluid through the brain interstitium; and C: clearance of fluid from the interstitium into perivenous, perineural and dural lymphatic spaces, again in part facilitated via AQP4 (6).

## Glymphatic system

The glymphatic system is an alternate pathway of the scavenger system in the brain, and studies indicate that it predates the development of the dural sinus drainage pathway. It facilitates the exchange of the metabolites between the perivascular space CSF and the brain. CSF movement is driven initially by the passive pressure gradients and subsequently by the dynamic exchange with the help of AQP4 channels, which are distributed across the brain (6–9).

## Venous drainage of the CSF

Arachnoid granulations (AG), extensions of the arachnoid matter, drain the CSF into the dural venous sinuses. However, alternate drainage pathway systems exist other than dural sinuses, including the perineural CSF drainage through the skull base into the cervical lymph nodes. Factors like arterial pulsations, sleep, and body position enhance this movement. Evidence suggests an alternate pathway for CSF drainage through the cribriform plate, along cranial nerves, into the nasal mucosa, and ultimately into nasal lymphatics (6–9).

Characterizing the pathophysiology of IIH poses a significant challenge in distinguishing primary causes from secondary effects. The pivotal role of increased intracranial pressure in IIH suggests that the primary event is a fluid imbalance and could potentially be secondary to physiological factors. The ensuing cascade of heightened pressure, coupled with changes in the enclosed space, appear to evolve into a self-perpetuating, relentless downhill progression. Furthermore, exploring epidemiological risk factors, notably obesity, and female sex, may offer valuable mechanistic insights into the development of IIH (1, 7). Explanations for the mechanisms underlying IIH have attempted to elucidate the pathological underpinnings by attributing them to one or all of the following: overproduction of CSF; impaired resorption of CSF; dysautoregulation of cerebral blood flow; dysregulation of fluid homeostasis leading to subtle white matter edema; dysregulation of AQP1 and AQP4 receptors; and proinflammatory states caused by mitochondrial dysregulation or circulating signalling leptins (1).

One of the common observations in the pathogenesis of IIH is a strong association with abnormalities in the venous outflow, and stenosis of the transverse sinus was seen in up to 93% of patients with IIH in one study (10); however, the cause-and-effect relationship has not been conclusively proven, and it has been shown in some studies that there is the reversibility of the sinus narrowing with effective medical therapy, and it is, therefore, possible that the venous stenosis maybe a result rather than the cause for the same (10). Conflicting evidence of persisting venous sinus stenosis despite the normalization of Intracranial Pressure (ICP) suggests that there is more than one mechanism at play. To resolve these contradictions, two postulations were put forth. (1) Non-Venogenic: An extrinsic compression of the dural venous sinus contributing to raised ICP and on venography this compression usually shows as a long tapering stenosis. This form of stenosis is usually reversible with normalized ICP and is likely associated with abnormal CSF production/absorption. (2) Venogenic: This is likely associated with intrinsic venous disease and associated stenosis and may be driven by underlying venous inflammation or anatomical variations. This Venogenic IIH may become symptomatic with precipitating causes such as venous stenosis or change in CSF dynamics. This venogenic IIH is likely less responsive to changes in the ICP (11).

## MR imaging and imaging correlates

Typical imaging observations in IIH are those of increased pressure in an enclosed space (Monro-Kelly Hypothesis) (12). The intracranial structures accommodate to compensate for the increased pressure by distributing the pressure with imaging manifestations such as empty sella, optic nerve protrusion and flattening of the posterior globe, distended optic nerve sheath, optic nerve tortuosity, and slit-like ventricles, which are all believed to be compensatory mechanisms to overcome the increased pressure. As alluded to above, transverse sinus stenosis is probably a secondary sign initially but potentially leads and participates in the cascade of downhill spiral of increased pressures.

## Empty sella

**Figure 1:**
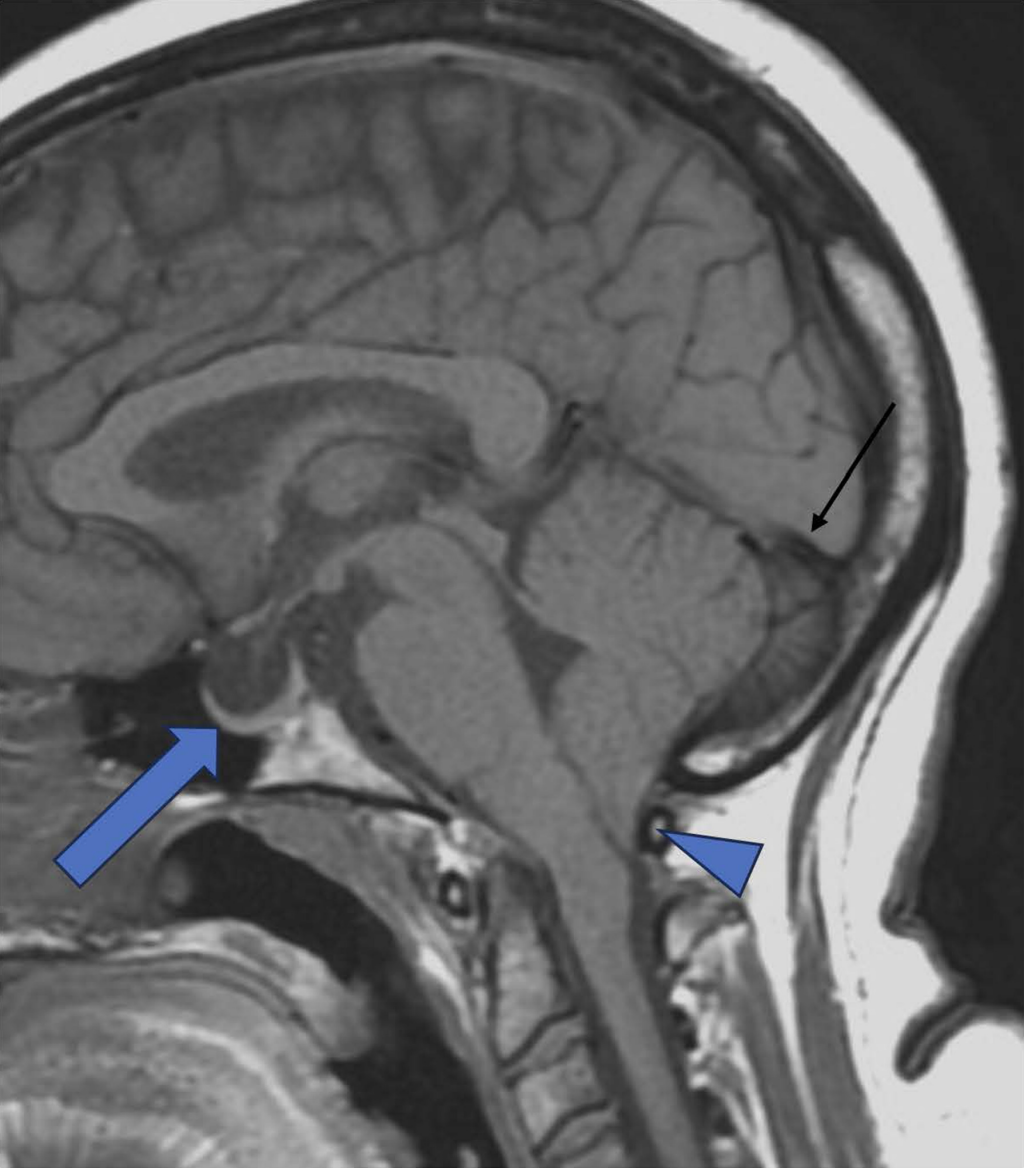
Empty sella. Sagittal T-1 TSE demonstrating Empty sella (Thick Arrow), Tonsillar herniation (Triangle), and concave Straight sinus (Black thin arow). (Tonsillar herniation can be associated with IIH, though rare).

The term “empty sella” (Figure 1) refers to a neuroimaging finding in which the sella turcica, the skull base structure that houses the pituitary gland, is either partially (partial empty sella) or fully filled with CSF (13). In the absence of the other causes that can result in the secondary empty sella (SES), primary empty sella (PES) is considered one of the most sensitive imaging findings associated with IIH. The postulated causes for the PES include dilated CSF spaces due to transmitted pressures from the IIH, resulting in dilated suprasellar cisterns and herniation of these cisterns into the sella, thus leading to PES. It was also theorized that the bony enlargement of the sella turcica was caused by chronic increased ICP, resulting in a larger and, therefore, proportionally more “empty” sella (13).

The typical measurements of sella turcica are between 4 and 12 mm in height and 5–16 mm in width. In IIH, sella was 38% greater in people than those without, with only a slight reduction in the pituitary gland size (14, 15). Patterson et al. observed that an MRI-measured pituitary-to-sella turcica ratio of <0.5 increased the likelihood of increased ICP^1^ (16). Partial empty sella has been found to be a highly specific finding of IIH (95.3%, *p* < 0.0001), but the absence of this finding does not rule out the diagnosis (17). The finding of the empty (or partially empty) sella is known to be reversible if there is an early intervention.

## Changes in the optic nerve

The optic nerve can show sheath distension, tortuosity over time, and, more specifically, disc protrusion. This triad of observations is thought to be linked directly to transmitted pressure from the IIH into the optic nerve sheath.

**Figure 2:**
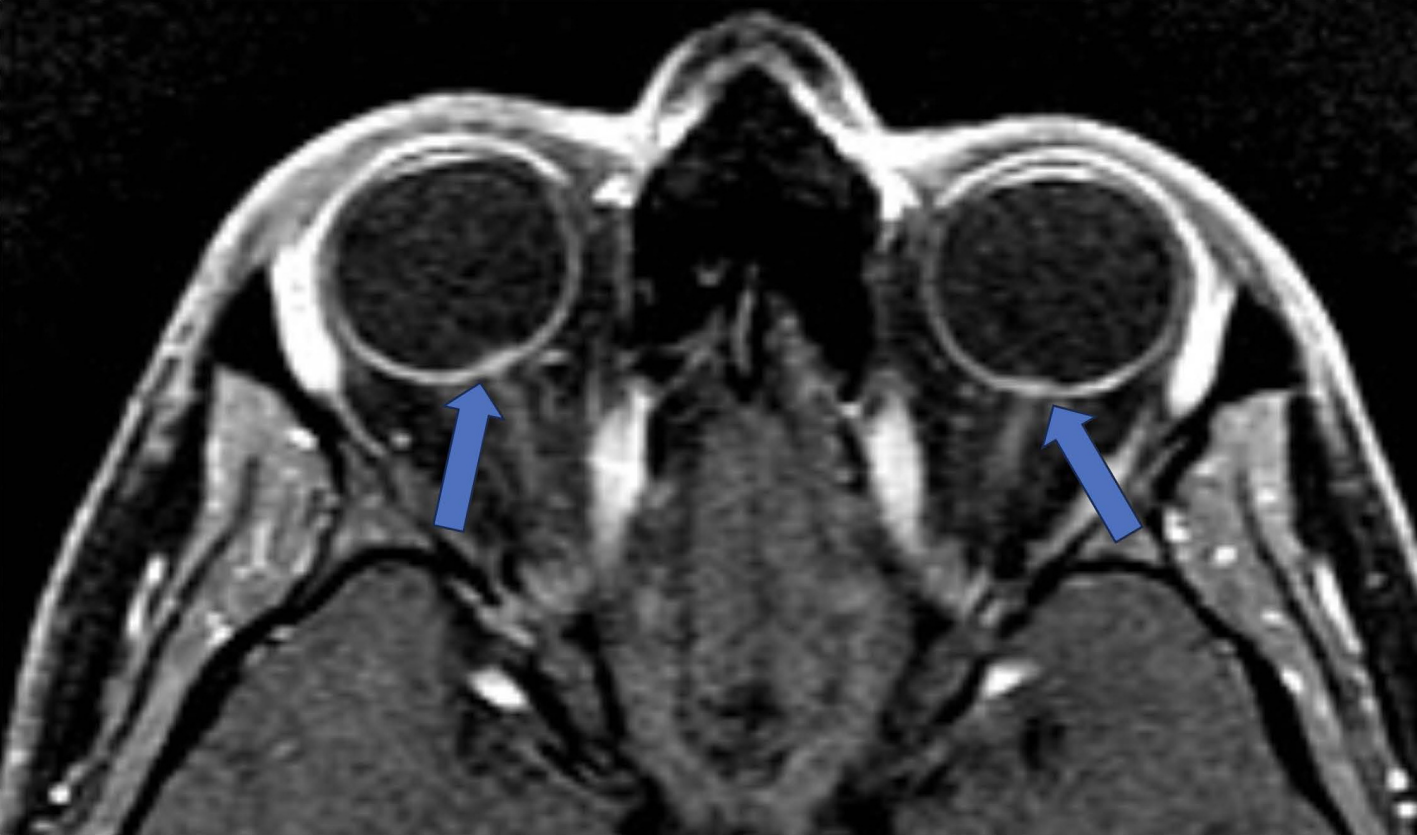
Protruded optic nerve and optic disc enhancement. T-1 Weighted fat saturated post contrast axial sequence through orbits: Enhancing optic discs with intraocular protrusion in the vitreous chamber of the globe (blue arrows).

**Protruded optic nerve and swelling** of the optic nerve head (Figure 2) are considered the clinical correlates of papilledema (18). Agid et al. found optic nerve protrusion to be present in 3.3% of people with IIH and 0% of controls (17).The protruded optic nerve head is associated with enhancement on the post-contrast T1w MR sequence and is a useful observation to identify disc edema. It is often restrictive (low signal) on diffusion-weighted imaging. This finding may appear earlier than the positive optical coherence tomography (OCT) and could serve as a biomarker for the papilledema risk in IIH (R = 0.74, *P* = .01) (19).

**Figure 3:**
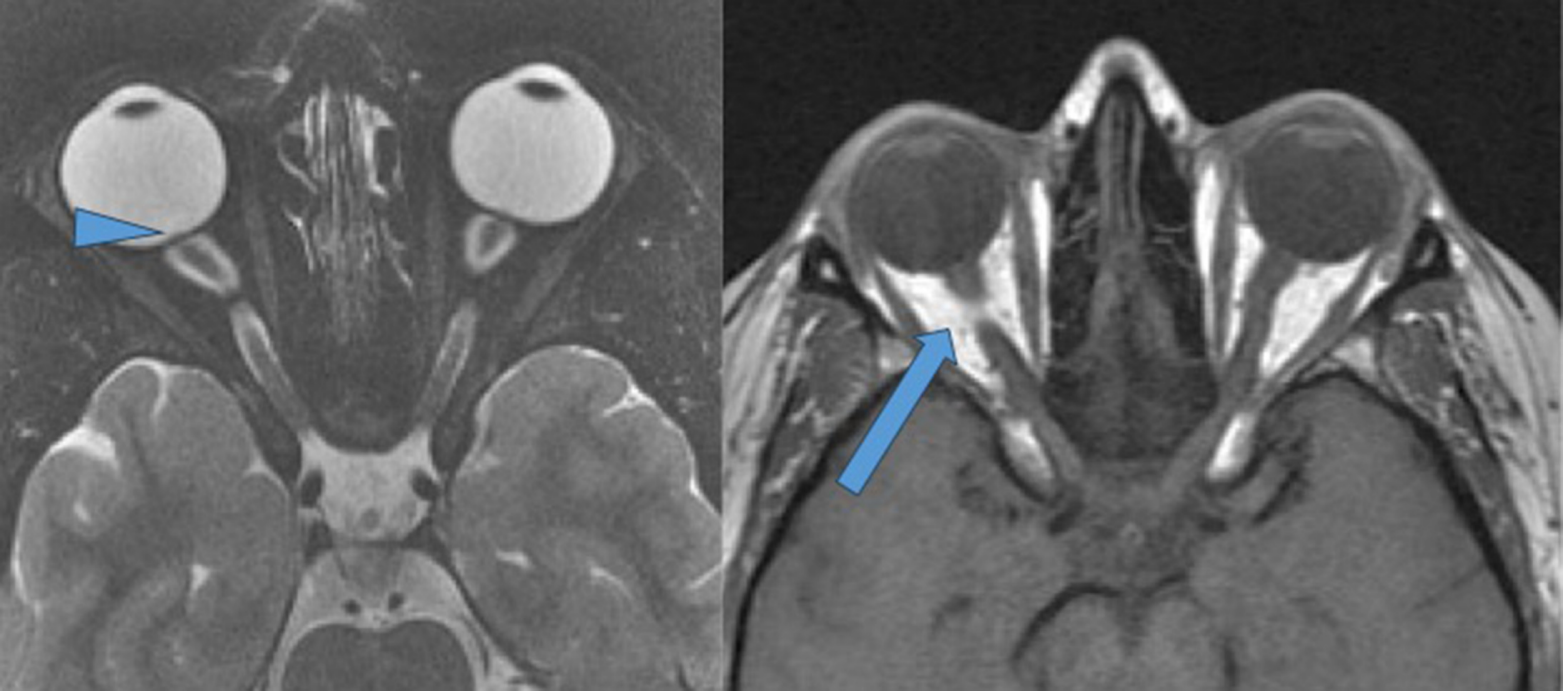
Changes in the globe: posterior globe flattening and tortuosity of the optic nerves. T-2 weighted fat saturated axial sequence through orbits demonstrating posterior globe flattening and tortuous intra-orbital segments of the optic nerves. Also note protruding optic nerve head (arrowhead). T-1 weighted spin echo axial of the orbits demonstrating “smeared fat sign” (blue arrow).

**Tortuosity of the optic nerve** (Figure 3) occurs due to the fixation of the nerve at proximal and distal points, and increased CSF pressures in the nerve make it tortuous. Detection of tortuosity depends on the MRI slice thickness and orientation, with vertical tortuosity being more specific for increased ICP than horizontal tortuosity. Vertical tortuosity of the ON is often accompanied by a “smear sign,” in which the midportion of the ON appears obscured by a “smear” of orbital fat on T1-weighted image (20). A meta-analysis of MRI signs in IIH by Kwee et al, has shown that tortuosity of the optic nerve is an observation with low sensitivity (40%) but high specificity (88.4%) (21).

**Figure 4:**
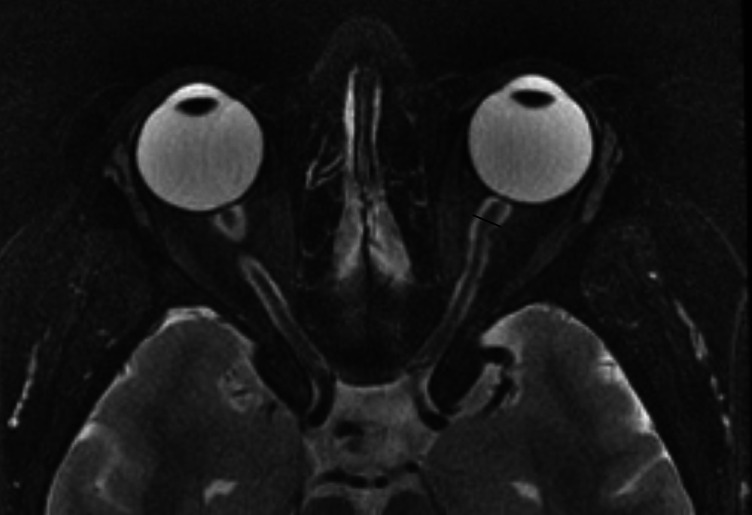
Distended optic nerve sheath diameter (ONSD). T-2 FAT SAT Axial Orbits: Dilated optic sheath >5.6 mm is considered abnormal (Specificity increases when the diameter is above 6 mm).

**Distended optic nerve sheath diameter (ONSD)**: Distension of the optic nerve sheath is one of the reliable signs of IIH (19, 21). The ONSD is measured approximately 3 mm posterior to the globe, where the sheath is at its most compliant and usually most dilated (22). The cross-sectional diameter of the optic nerve sheath in normal individuals is considered to be up to 4.5 mm (Figure 4). A study by Hoffman showed that a diameter between 5.5–5.6 mm has been associated with 72% sensitivity and 80% specificity (23). Agid et al. demonstrated this finding in up to 68% of patients with IIH (17). Caglayan et al. have shown improvement in the diameter (reduction) after therapeutic intervention, but it is still higher than normal controls (24). Cross-sectional diameter of subarachnoid space thickness is also used as a measure of dilatation of the ONSD, and, when it is more than 2 mm, it is considered abnormal.

## Changes in the globe: posterior globe flattening

The typical posterior convexity contour change of the globe is likely attributable to increased cerebrospinal fluid (CSF) pressure transmitted through the optic nerve sheath (ONS) to the eyeball. This transmitted pressure is also implicated in the flattening of the optic disc and the inward protrusion and enhancement of the optic nerve papilla. Posterior globe flattening (Figure 2) refers to the straightening out of the curvature of the posterior sclera in the region where the sclera attaches to the optic nerve (17). This sign is shown to be present in roughly 50% of people with IIH, often a subjective assessment (13). However, a quantitative method developed by Alperin et al. showed that this observation has a very high sensitivity [control and IIH groups (0.93 ± 0.020 vs. 0.91 ± 0.022 (*P* = .003) (19) (Figure 2).

## Changes in ventricular size: slit-like lateral ventricles

Narrowing and collapse of the ventricles, referred to as slit ventricles, was described as a sign of IIH and was described in the past when CT was the primary imaging modality of choice and was linked to the increased parenchymal edema and had a low sensitivity as a maker of IIH (13, 25).

## Meningoceles

**Figure 5:**
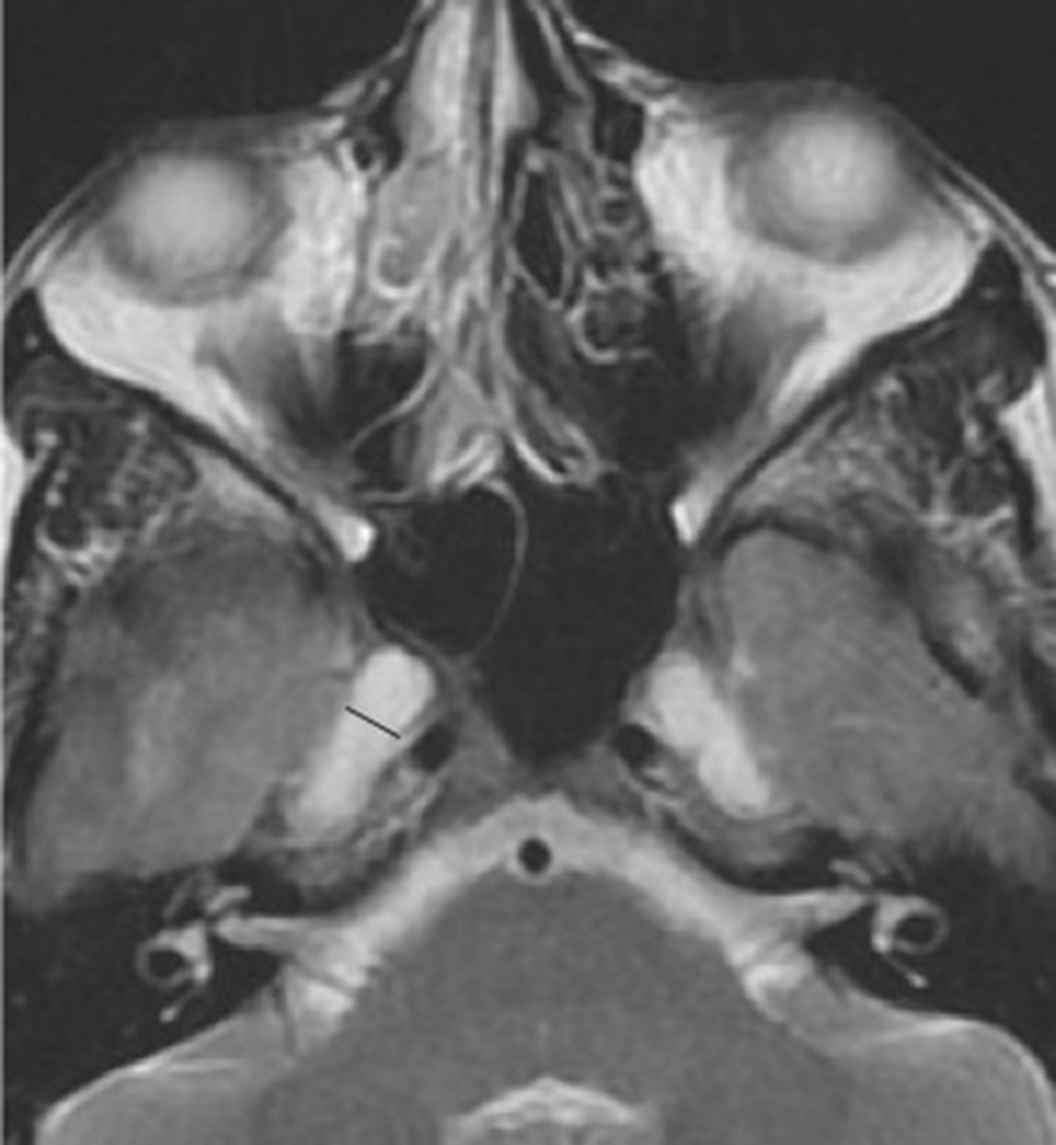
Pseudomeningocele. T-2 weighted axial sequence through orbits demonstrating pseudo arachnoid diverticula commonly noted at Meckel's cave (black line) but can be visualized at multiple levels at the basal foramen.

Meningoceles at the petrous apex and prominent Meckel's caves are specific signs of IIH, but less sensitive (23, 26). Expansion of the CSF spaces can be found around the neural exit foramen, such as surrounding the oculomotor nerve as it courses through the parasellar space, the abducens nerve as it courses through Dorello's canal, the facial nerve at the geniculate fossa, and the hypoglossal nerve at the hypoglossal canal are noticeable with markedly elevated intracranial pressures (22, 27, 28) (Figure 5).

## Transverse venous Sinus stenosis

**Figure 6:**
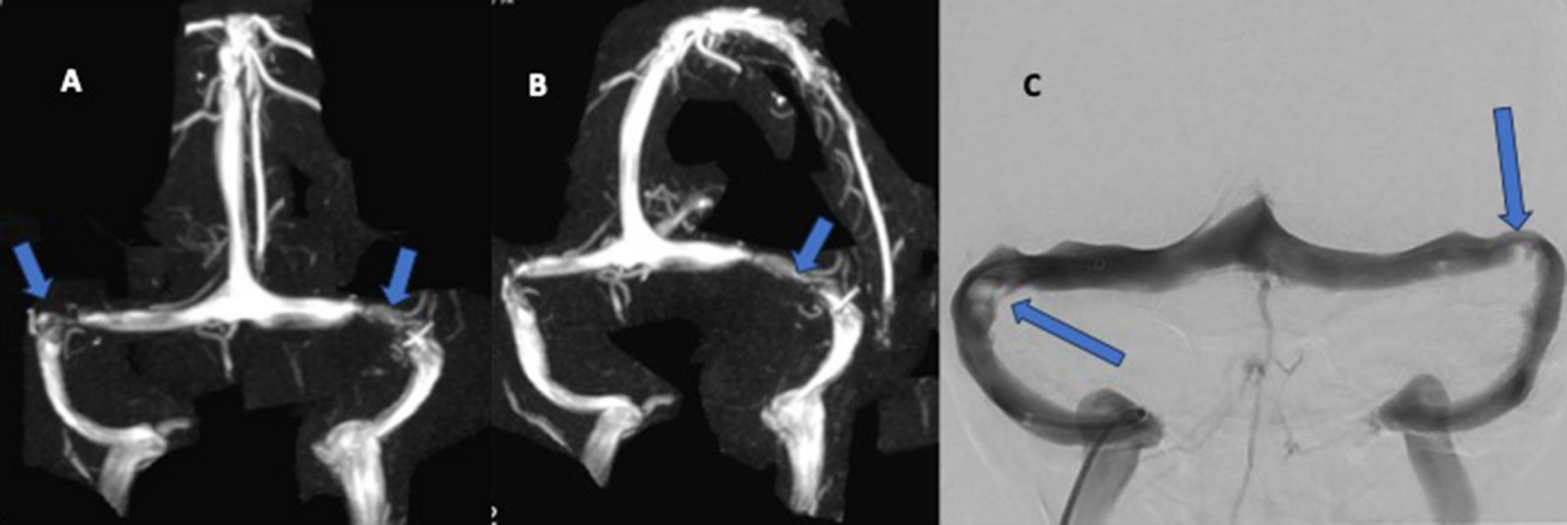
**(A–C)**: transverse venous sinus stenosis. **(A,B)**: 2D time of flight (TOF) MRV with MIP reconstructions in the frontal and oblique rotations demonstrating stenosis of the transverse sinuses bilaterally possibly from hypertrophied arachnoid granulations on the right side and likely inﬂammatory narrowing on the left side (focal irregular narrowing) (Arrows showing area of narrowing). **(C)**: DSA venography of the different patient showing similar narrowing of the left transverse sinus and arachnoid granulations on the right.

Bilateral transverse sinus stenosis is shown to be associated with IIH in 90% of the subjects (Figures 6A–C). Using gadolinium bolused MRV sequence, bilateral transverse sinus (TS) stenosis of >50% degree is seen in 93% of patients with IIH, suggesting that this is the most sensitive imaging to identify as well as grade the stenosis (29). In another study that has looked at the role of CE MRV in a stenting cohort, Boddu SR et al. reported 100% sensitivity and 100% negative predictive value for the detection of recurrent dural venous sinus stenosis (29). Measurement of the transverse sinus stenosis using non-contrast 2D TOF MRV has several pitfalls, such as in-plane signal dephasing and inability to detect and differentiate slow flow from absent flow, and perhaps to be avoided (30). Recent studies have shown contrast-enhanced 3D volume T-1 sequences and reconstruction aid in characterizing the percentage of venous sinus stenosis in addition to morphological changes such as exaggerated concavity in the dural sinuses (27, 31). Phase contrast MRV does not suffer from the inflow artefacts like in 2DT0F and has superior contrast resolution (32). However, the disadvantages include higher time to acquire (usually over three minutes) and aliasing if the velocity encoding steps are not properly tailored (Figures 6A,B).

**Vessel wall Imaging**: There is limited information on vessel wall imaging applications in the assessment of dural venous sinuses. Intracranial vessel wall imaging has been shown to be effective in identifying arterial vasculopathy. Quan et al, showed that contrast-enhanced IVW is more accurate compared with PC-MRV in assessing stenosis degree in IIH patients in 62 patients with suspected IIH (33). Yang et al. compared imaging characteristics of thrombus on non-contrast 3D T 1-weighted IVW between patients with early subacute and late subacute cerebral venous thrombus. IVW accurately identified 113/116 segments having venous thrombus (sensitivity 97.4%) and accurately depicted thrombus volume (34). Intradural sinus webs are possibly best resolved using dark blood studies (35).

## Advanced imaging techniques

**Figure 7:**
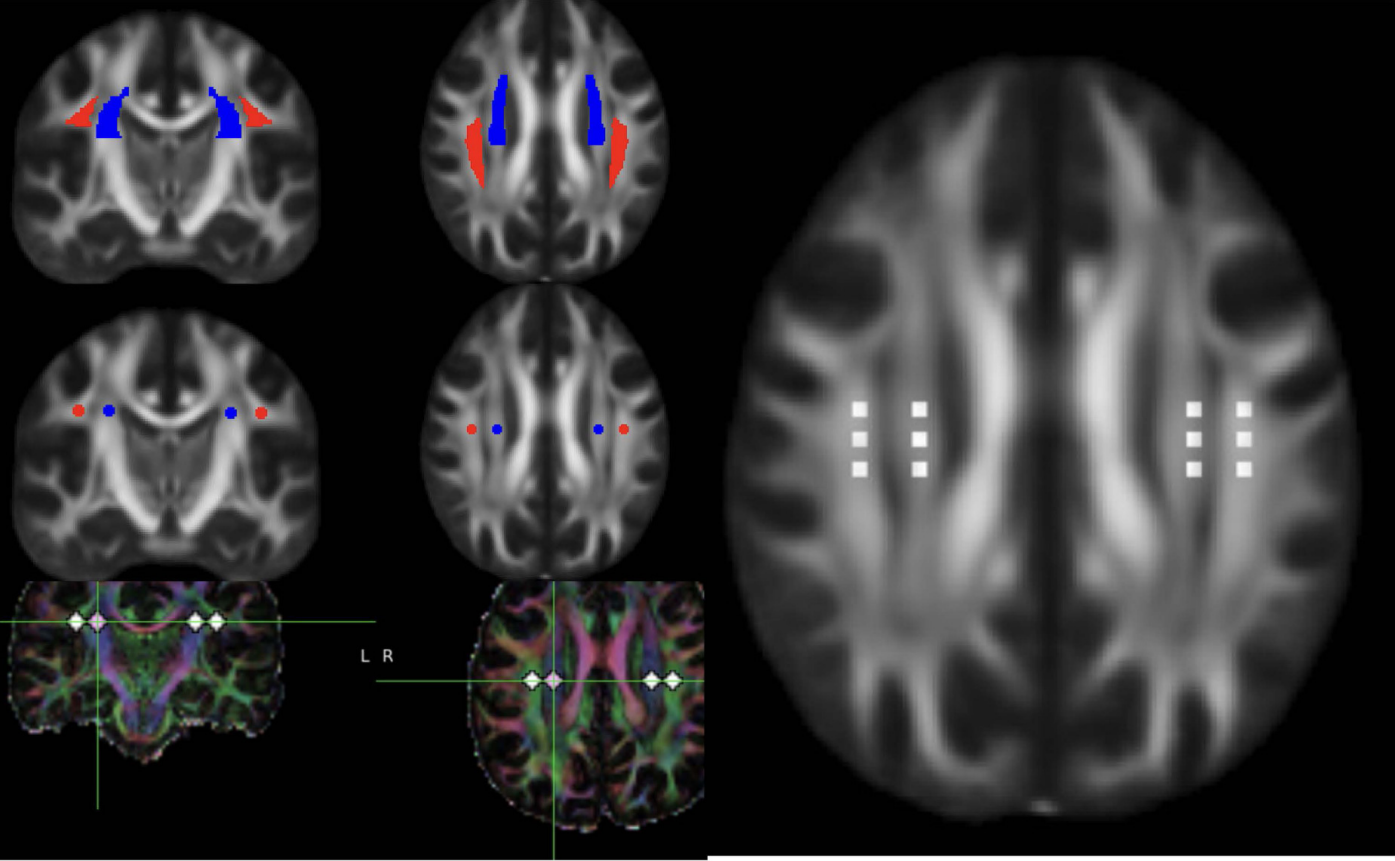
DTI-ALPS (diffusion tensor image analysis aLong the perivascular space. DTI data in axial and reconstructed coronal planes showing the ROI in the projection and commissural fibers to measure the ALPS ratio. Please note the ROI are placed using atlas based co registered image as well as manually.

Although several models have been formed to explain the drainage pathways of the CSF, the most commonly used model for neurofluid circulation is the Glymphatic system, which will be briefly discussed to explain the pathophysiological underpinnings of the IIH (Figure 7).

The concept of perivascular space serving as a conduit that drains the CSF into the interstitial space of the brain parenchyma and subsequently collects the waste products back into the perivascular space around the veins before being drained out of the brain was first introduced by Iliff et al. in 2012 (36). This pathway which combines the role of glial and lymphatics to clear the waste products of the interstitial space, was termed a glymphatic system (37).

While the initial forces that guide the CSF into the perivascular space of this conduit include arterial pulsation and convective bulk flow of ISF, subsequently, CSF entry into the interstitium is through more dynamic forces facilitated by the AQP4 water channels distributed in the foot processes of astrocytes that constitute the outer wall of the perivascular space (6).

The glymphatic system is a whole-brain perivascular network, which promotes CSF/interstitial fluid exchange. Imaging the abnormalities leading to abnormal drainage by the glymphatic system was explored at several levels. Advanced MRI methods such as perivascular space volume fraction, fractional volume of free water in white matter (i.e., brain interstitial fluid) from a bi-tensor diffusion tensor imaging model and index of diffusivity along the perivascular space (ALPS index) are being explored to measure the myelin water fraction and interstitial fluid volume (38, 39). Multi-shell diffusion tensor imaging, Intravoxel incoherent motion (40), and multi-echo ASL (41) (blood-brain interface water permeability by calculating the exchange time of magnetically labeled intravascular water across the blood-brain interface.) are few other techniques that are likely to be used to measure the extracellular water and blood brain barrier disruption without the necessity of intravenous gadolinium.

Existing evidence indicates that that the neuroinflammation is present in the patients with IIH (42). The defining response of the brain to neuroinflammation is increased capillary permeability and disruption of the blood brain barrier. In IIH, there is both increased production and decreased absorption of the neurofluids and both leading to increased perivascular space fluid volume (43). Taken together these two important pathophysiological events may have cause and response interlink and advanced imaging algorithms can be tailored to address these pathophysiological pivots. Dynamic Contrast enhanced perfusion metric K trans is a sensitive technique for measuring blood-brain barrier integrity (44). This technique has been extensively used in several conditions associated with abnormal glymphatic pathways and could potentially play an important role in understanding the role of the in IIH. Diffusion Tensor Image Analysis ALong the Perivascular Space, a new method to measure the interstitial fluid and CSF imbalance is increasingly used by several groups to understand the pathophysiology of IIH. Application of these advanced MR imaging algorithms are likely to play a crucial role particularly in the measurement of treatment response.

## Conclusion

The pathophysiological basis of IIH is still unknown. However, there is potential for gaining clarity in understanding the pathophysiological underpinnings through advanced MR imaging techniques. This, combined with robust anatomical imaging, can assist both in the diagnosis and the decision-making in the management and follow-up.
